# Supplementary material for: Are carbon emissions trading and green financial instruments synergistic? -Comprehensive quantitative research based on content analysis
Source: PLoS One. 2024 Mar 7;19(3):e0298601. doi: 10.1371/journal.pone.0298601 (PMC10919601; doi:10.1371/journal.pone.0298601)
Supplement: S1 Data — (ZIP) [file pone.0298601.s001.zip › Data/地级市Z_F工作报告.zip!/╣π╬≈/╙±┴╓╩╨/└┤╫╘╣1⁄2╓┌║┼í╛┬φ┐╦╩2╛▌═°í┐_2017.docx]

各位代表：

现在，我代表市人民政府向大会作政府工作报告，请予审议，并请市政协委员和列席会议的同志提出意见。

一、2016年工作回顾

过去的一年，面对复杂严峻的发展环境，在自治区党委、政府以及市委的正确领导下，在市人大及其常委会、市政协的监督支持下，我们坚持稳中求进工作总基调，牢固树立发展新理念，主动适应经济发展新常态，积极融入国家“一带一路”战略和自治区“双核驱动、三区统筹”发展格局，深入实施“东靠南下、通江达海”发展战略，大力推进“四大攻坚”“五大战略”，认真开展“三个年一活动”，扎实做好改革发展稳定各项工作，经济社会实现持续健康发展，实现了“十三五”良好开局。

——经济运行更加稳健。经济基本面保持平稳，呈现缓中趋稳、稳中向好态势。初步预计，实现GDP1550亿元，增长8%；财政收入149亿元，增长6.7%；固定资产投资1472亿元，增长10.5%；规模以上工业增加值470亿元，增长8.7%；社会消费品零售总额662亿元，增长10.2%。有12项主要经济指标增速高于全区平均水平。

——经济结构更加合理。产业结构、投资结构、消费结构进一步优化，三产比重提高1.3个百分点，产业结构优化为17.9：42.7：39.4。新材料、新能源、生物医药等新兴产业增速高于规模以上工业增速。民间投资占固定资产投资比重提高到83%。服务业对经济增长的贡献率达39%。

——发展动力更加强劲。供给侧结构性改革效应初显，工业增加值增速高于全区1.1个百分点，规模工业利润总额增长10.8%。“双创”活力不断增强，新增市场主体2.7万户，增长16.6%。中金二期炼钢车间建成投产。玉林内燃机产业基地获认定为第一批国家火炬特色产业基地。

——基础设施更加完善。“修机场、建码头、接高铁”三件大事取得重大突破，完成黎湛铁路贵港至玉林段电气化改造，建成玉林火车站新站房，实现开通动车；开工建设玉林民用机场试验段工程、玉林至湛江高速公路、荔浦至玉林高速公路、松旺至铁山港东岸高速公路。园博园建成开放，成功承办第八届广西园林园艺博览会。被评为“宽带中国”示范城市。

——人民生活更加殷实。全市财政民生支出251.7亿元，增长12.6%，占一般公共预算支出的79.1%。居民人均可支配收入20726元，增长9%。“六大脱贫攻坚工程”深入推进，实现10.2万贫困人口脱贫、111个贫困村出列、陆川县脱贫摘帽的目标。

一年来，我们主要抓了以下工作：

（一）大力推动经济稳定增长。坚持把稳增长摆在首要位置，以推进工业、投资、消费、农业、进出口、财政“六稳”为重点，制订出台降低企业成本35条、扶持发展规模以上工业企业等11项稳增长措施，开展工业稳增长八大行动，扶持发展总部经济、品牌经济，推动企业上规、上限、上亿、上市，全面打好稳增长攻坚战。降低企业成本12亿元；成立玉林市中小企业融资性担保有限公司，全市中小企业担保贷款余额20.4亿元；新增新三板挂牌企业4家；新增规模以上工业企业28家、亿元工业企业9家；新增广西著名商标13个、中国驰名商标1个、广西名牌产品企业7家、广西服务业品牌企业4家；中金公司、兴鹏鞋业等6家企业总部落户玉林。

（二）大力推进重大项目建设。完善重大项目推进机制，突破项目审批、征拆、融资瓶颈制约。重点推进77个自治区统筹项目、12个西江经济带基础设施建设大会战项目和568个市统筹项目。玉柴铸造中心三期、玉柴船用柴油发动机、玉药提取等重大产业项目建成投产，中滔环保产业园、中金热轧项目、玉柴气体发动机、康美、和黄等项目加快建设，郁江引水工程、容县杨村风电场二期、罗田水库供水工程等重大基础设施项目即将竣工。北流塘岸至玉林新桥二级公路建成通车，G324国道大修工程、贵港木格至博白水鸣、岑溪南渡至陆川二级公路建设加快推进，陆川至浦北二级公路开工建设。争取上级债券、专项资金127.8亿元；新增贷款163亿元，增长21%，增幅排全区第一，创历史新高，金融机构贷款余额突破1000亿元，社会融资总量285亿元。清除整治“两违”42.4万平方米。

（三）大力推进产业转型升级。突出产业结构调整，加快构建现代产业体系。完成园区基础设施投资25.5亿元。新开工建设工业项目45个，竣工50个。新增国家级高新技术企业4家。开工建设玉林汽车新城、健康小镇、弘润综合农贸市场等重大商贸项目。限额以上网上批发和零售额增长50%，快递业务量增长133.8%。实施旅游项目129个，容县成功创建广西特色旅游名县，云天文化城、容州古城、都峤山风景区成功创建国家4A级景区，新创四星级乡村旅游区、农家乐12个，广西休闲农业与乡村旅游示范点5个，旅游总消费达281.6亿元，增长41.3%。创建自治区级现代特色农业（核心）示范区3个，新增农业龙头企业24家、农民专业合作社800户、家庭农场258家，发展农业电商260家，茂林镇鹿塘社区荣获“中国电商旅游第一村”和“中国美丽休闲乡村”称号。全面推广“高架网床+益生菌”养殖模式，推动养殖产业转型发展。

（四）大力提升城市功能品位。实施市政公用设施项目213个，加快城市道路、园林、供水、供电、污水处理等基础设施建设，清宁路、金港大道、江南大道全线通车，完成教育东路、教育中路改造提升，火车站站前广场改造加快推进，万花楼重建项目实现开工。开展市容“五乱”和“四车”综合整治活动，创建人民路等样板街，市容市貌有效改观。创建文明城市、智慧城市、海绵城市工作扎实推进。加快玉林北流同城化发展。实施大县城战略，支持重点镇和中心镇建设发展，9个乡镇入选广西百镇建设示范工程。全市城镇化率达47.5%。城市建成区绿化覆盖率37.1%，人均公园绿地面积9.6平方米。

（五）大力深化改革开放合作。深化供给侧结构性改革，去产能、去库存、去杠杆、降成本、补短板有效推进。全国农村改革试验区建设、北流市农村集体经营性建设用地入市和土地征收制度改革试点工作稳步推进，3项试验成果实现了国家层面的政策转化。深化“先照后证”“六证合一”“双随机、一公开”“全城通、就近办”等商事制度改革，推进项目审批预审查“化零为整”试点。不动产登记工作扎实推进。加快推进中泰（玉林）旅游文化产业园、中国—文莱（玉林）健康产业园等中外合作项目。成功举办第16届国际传统药物学大会、第八届药博会、第六届陶博会和2016年海外华侨华人玉林恳亲大会，被评为2016年度中国优秀会展城市，药博会、陶博会被评为中国十佳优秀特色展会。深入实施加工贸易倍增计划，加工贸易占全市进出口总额达47%。招商引资新落地项目292个，累计到位资金565亿元。

（六）大力推进美丽玉林建设。深入开展“美丽玉林·生态乡村”活动，投资21.4亿元，实施“六个一”工程，创建2个自治区级生态镇、79个生态村。玉东新区茂林镇被授予“中国美丽乡村建设示范镇”称号。完成8361个村屯绿化。植树造林27.9万亩。启动玉林—北流—容县生态景观带、九洲江生态景观带、南流江百里景观长廊等生态项目建设，全面提升“五彩玉林·田园都市”品牌。抓好中央环境保护督察突出问题整改工作，大力开展以治水、治气、治渣为重点的环保专项行动，城乡环境质量稳中趋好。全面深化重点流域整治，九洲江、南流江水质明显提升。

（七）大力推进社会治理创新。深入推进“三官一律”进村入社。完成160个城乡社区规范化建设。加大全民法治宣传教育力度。加快信用体系建设，创建信用乡镇24个、信用村342个。创新矛盾纠纷综合调处机制，加快信访网络信息平台建设，最大限度把矛盾纠纷化解在基层、解决在萌芽状态。开展食品药品安全专项行动，食品药品安全监管能力明显提升。加强安全生产监管，安全生产事故起数和死亡人数双下降。强化火灾隐患整治，消防安全持续稳定。加强社会治安防控，抓好重点领域、重大项目和园区治安环境整治，强化反恐维稳工作，严厉打击各类违法犯罪行为，刑事案件呈下降趋势，社会治安总体向好，群众安全感满意度明显提升。

（八）大力抓好民生事业发展。全面完成为民办实事工程项目。建成保障房4200套。完成农村危房改造8421户。建设水库移民新村项目51个。实施农机补贴2680万元。建设村级公共文化服务中心121个，成功创建国家公共文化服务体系示范区。全民健身活动深入开展。建成市青年学习实践与发展活动中心、妇女儿童活动中心，创建村（社区）儿童家园251个。新建玉州区金港小学、城南实验小学等一批中小学校，大幅度增加学位供给，有效化解“大班额”“入学难”问题，北流市通过义务教育均衡发展国家督导评估，玉州区、容县通过自治区评估。建设创业孵化基地13家，新增城镇就业3万人、农村劳动力转移就业5万人，城镇登记失业率3.3%。居民消费价格指数累计上涨2.4%。开工建设广西医科大学玉林校区，城市公立医院改革不断深化，城乡公共卫生服务水平稳步提高。全面两孩政策平稳实施，计生事业稳步发展。社会保障体系不断健全，城乡居民养老保险、基本医疗保险和城镇居民大病保险制度日益完善。发放低保资金4.2亿元，城乡低保对象全部实现应保尽保、按标施保。

国防教育和国防后备力量建设不断加强，驻玉部队在支持地方建设上做出重要贡献，军政军民团结局面进一步巩固，实现再创“全国双拥模范城”。中直、区直驻玉各单位为经济社会发展提供大力支持。民族、宗教、外事、侨务、港澳台事务、人防、统计、供销、地震、气象、水文、无线电管理、方志、档案、机构编制、住房公积金管理、老龄、残疾人、科普等工作取得新成绩。工会、共青团、妇联等群团组织重要作用得到进一步发挥。

一年来，我们深入开展“两学一做”学习教育，认真贯彻落实党的十八届六中全会精神，坚持从严治党，切实加强政府自身建设。坚决贯彻执行市委的决策部署，认真执行市人大及其常委会的决议、决定，自觉接受市人大及其常委会、市政协和社会监督，认真听取各民主党派的意见，人大代表建议和政协委员提案全部办结。坚持依法行政和科学决策，加强政务服务体系建设，机关作风进一步好转。大力推进行政审批制度改革，做好“放、管、服”工作，取消、调整54项行政审批事项。认真履行党风廉政建设“两个责任”，锲而不舍落实中央“八项规定”精神，常态化开展查处发生在群众身边的“四风”和腐败问题专项工作。行政监察和审计监督进一步加强。政务公开和信息公开不断深化。

各位代表，过去一年的成绩来之不易。这是自治区党委、政府和市委坚强领导的结果，是市人大及其常委会依法监督的结果，是市政协和社会各界监督支持的结果，是全市人民团结奋斗的结果。在此，我代表市人民政府，向全市人民，向人大代表、政协委员，向中央及自治区直属驻玉单位、驻玉部队、武警官兵，向各民主党派、工商联、无党派人士、各人民团体和海内外所有关心、支持玉林发展的各界人士，表示崇高的敬意和衷心的感谢！

在看到成绩的同时，我们也清醒地看到，我市发展中仍存在不少困难和问题，主要是：实体经济发展困难，部分经济指标没有达到预期，稳增长难度较大；产业转型升级步伐不快，产业结构不合理，经济发展的新动能不足；项目建设要素制约突出，缺乏重大项目支撑，投资持续增长后劲不足；城乡基础设施不完善，现代综合交通网络不健全，生态体系建设任重道远；脱贫攻坚任务艰巨，保障和改善民生的压力较大；政府工作还存在不足，一些地方和部门的服务意识不强、行政效能不高，少数公务人员甚至违法违纪，严重影响了党和政府的形象。我们将坚持问题导向，采取有力措施，加快补齐短板，让全市人民感受到实实在在的变化和成效。

二、2017年工作总体要求和目标任务

2017年是实施“十三五”规划的重要一年，是供给侧结构性改革的深化之年。当前我国经济运行总体平稳，国家确立了适应经济发展新常态的经济政策框架，引导经济朝着更高质量、更有效率、更可持续的方向发展。自治区党委提出营造“三大生态”、实现“两个建成”，谱写建党百年广西发展新篇章的宏伟目标，为我们带来了重大发展机遇。我市经济转型步伐进一步加快，产业集聚效应不断加强，基础设施不断完善，发展新动能加快形成。只要我们抢抓机遇，主动作为，扎实工作，就一定能够开创玉林转型跨越发展的新局面。

今年经济社会发展的总体要求是：全面贯彻党的十八大和十八届三中、四中、五中、六中全会精神，深入贯彻习近平总书记系列重要讲话精神和治国理政新理念新思想新战略，紧紧围绕自治区党委、政府和市委重大部署，按照“五位一体”总体布局和“四个全面”战略布局，坚持稳中求进工作总基调，牢固树立和贯彻落实新发展理念，适应把握引领经济发展新常态，深化供给侧结构性改革，深入实施“东靠南下、通江达海”开放战略，大力推进“四大攻坚”“五大战略”，以提高发展质量和效益为中心，守住民生、环保两个底线，抓住投资、财政、实体经济三个关键，优化产业、工业、投资、消费四个结构，努力筑牢新基础、实现新突破，为加快实现“两个建成”宏伟目标打下坚实基础。

经济社会发展主要预期目标是：地区生产总值增长7.5%；财政收入增长5%；固定资产投资增长12%；规模以上工业增加值增长8%；社会消费品零售总额增长10%；外贸进出口总额增长6%；居民人均可支配收入增长9%；节能减排完成自治区下达任务；居民消费价格涨幅控制在3%左右；城镇登记失业率控制在4%以内。

实现以上要求和目标，需要敢于担当、奋勇向前的精气神；需要凝心聚力、团结拼搏的齐心力；需要开拓创新、攻坚克难的拼命劲。我们要坚定不移朝着目标奋勇前进，全力以赴把玉林的事情办好，决不辜负各位代表和全市人民的期望。

三、2017年重点工作安排

（一）实施创新驱动发展战略，培育经济发展新动力。

强化企业自主创新。大力培育发展高新技术企业、科技型中小企业，新增国家高新技术企业6家。支持企业引进和开发新产品、新技术、新工艺，加快改造提升传统产业。开展科技成果转化大行动，引进转移科技成果产业化项目20项以上。深入实施“质量强玉”战略，鼓励企业争创中国驰名商标和广西著名商标，大力推进品牌建设，培育发展新增长点。

加强创新平台建设。加快玉林高新区建设，建成科技企业孵化大楼，打造国家级孵化器。推进国家火炬计划内燃机特色产业基地建设，加快产业创新发展。深化与国家科研院所、高校等研发机构合作，组织实施一批产学研协同创新项目。加快国家内燃机检验检测中心、国家陶瓷重点实验室等一批研发平台、重点实验室建设。加快军民科技协同创新，促进国防科技成果转化。

激发创新创造活力。实施新一轮发明专利双倍增计划，促进专利申请总量和质量稳步提高。构建高新区、科技企业孵化器、大学科技园、大学生创业基地、创客空间等多方协同的创业创新机制，进一步激发广大科技人员的积极性和创造性。深入实施“人才强市”战略，建立创新型人才激励机制，依托重大项目和科研创新平台，培养和引进一批年轻有活力、创新能力强的团队骨干和中坚力量，建设一支结构合理、素质优良的创新人才队伍。

（二）发展壮大实体经济，厚植产业发展新优势。

加大实体企业支持力度。设立产业发展基金、政府性融资担保公司、农业担保公司，加大政府扶持、政策倾斜力度，引导社会资金向实体经济集聚，促进各种资源要素向实体企业配置。推进企业“退二进三”“退城进园”，促进产业集聚发展。推动装配式建筑与现代绿色建材产业基地、碳酸钙及新材料产业项目落地建设，大力推进陶瓷小镇建设，支持中金、银亿、康臣、海螺、三环、宏邦、亮亮等龙头企业做大做强。开展降低企业成本行动，帮助企业正税清费，降低企业运行成本。完善企业投融资平台，创建专项担保基金，设立企业贷款周转金，落实小微企业无还本续贷政策，努力解决企业融资难问题。优化对企业的服务，简化办事流程。大力推动企业上规、上限、上亿、上市，培育发展总部经济，新增规模以上工业企业20家、限上企业30家、亿元企业10家、上市（挂牌）企业5家，发展总部企业5家。

实施工业振兴计划。开工建设重大工业项目50个，竣工50个，增添工业发展新动能。支持玉柴发展壮大发动机和新能源汽车产业，打造国内先进的新型装备制造基地。建成中金二期冷轧项目，大力引进不锈钢深加工企业，提升有色金属产业。加快康美药业、康臣－玉药等中医药产业项目建设，培育壮大中医药健康产业。大力引进稀土等新材料精深加工企业，加快新材料生态产业园建设，积极发展新材料产业。推进华电分布式能源、光伏发电、风电等项目，加快发展新能源产业。推进龙潭进口再生资源园区“城市矿产”示范基地建设，积极发展再生资源产业。推动黑五类、五丰机械等企业新产品产业化。创新园区投融资和管理运营机制，完成园区基础设施建设投资25亿元以上，园区工业总产值增长10%以上。

促进服务业提质增效。抓好现代服务业集聚区建设，积极推进金城广场、奥园广场、交通物流园等项目，支持银丰国际中药港、宏进农批市场等专业市场做大做强，促进商贸物流业提质升级。新建、改建一批农贸市场，优化城乡市场布局。大力发展健康养老、商贸餐饮、文体娱乐、教育培训等服务业。实施“互联网+”计划，大力培育发展电商，加快发展分享经济、数字经济、创意经济等新业态新模式。办好玉博会暨中国－东盟小商品交易会、药博会、陶博会，大力提升会展经济。继续办好“岭南都会”消费购物节，支持举办房产、汽车、家居建材、农林产品等专业性展销会，活跃城乡市场。

发展壮大现代农业。推进农业供给侧结构性改革，着力增加 绿色优质农产品供给。加快现代特色农业示范区建设，力争建成自治区级示范区2个、县级4个、乡级7个，示范区经营总收入达10亿元。建设广西粮食主产区和优质稻高产示范区，确保粮食总产稳定在182万吨以上。推广马铃薯种植20万亩，打造南方马铃薯基地。积极发展有机富硒农业。大力发展粮食、水果、中药材、畜禽、水产等特色农产品及精深加工，培育智慧农业、电商农业、休闲农业等新型业态，推动农村一二三产业融合发展。加大家庭农场、农民合作社、农业龙头企业培育力度，培育50个农民合作社示范社、50家示范性家庭农场。

大力发展县域经济。深化县域体制机制改革，进一步加大简政放权力度，增强县域经济发展活力和动力。加快推进新型工业化、信息化、城镇化、农业现代化深度融合，坚持产业兴县、特色强县，引导县域错位发展、多元发展。启动实施一批县域经济突破工程，努力争创自治区工业大县、农业强县、旅游名县、生态美县、开放活县。支持小城镇发挥产业优势，加快发展健康养老、中药材种植、农林产业、日用陶瓷、休闲旅游等特色产业，打造一批产业、文化、旅游“三位一体”的特色经济强镇。

（三）加快生态旅游品牌建设，推动旅游产业发展新跨越。

打造城市旅游形象。深入开展“旅游文化年”活动，全力塑造“五彩玉林·田园都市”的城市旅游形象。加快桂东南旅游组织中心（玉林中心城区）、南流江“海上丝绸之路”旅游发展带、南广旅游发展带、环都市健康养生旅游片区、生态山水休闲旅游片区、客家温泉文化旅游片区建设，构建“一心两带三片区”的旅游发展格局。推进“五彩田园”、云天文化、大容山、贵妃故里、六万大山、客家温泉养生六大旅游集聚区建设，推动“景区旅游”向“全域旅游”发展，创建国家“全域旅游”示范市。

提升景区档次。加快推进福达文化旅游科技体验园、都峤山、龙珠湖等总投资190多亿元的30个旅游项目建设，建成一批精品景区。深入开展创特、创A、创星工作，巩固提升容县创特成果，支持北流创建广西特色旅游名县，大力推进“五彩田园”创建国家5A级景区，新创4A级景区3家、四星级乡村旅游区8家、四星级农家乐8家，加强乡村旅游区建设。积极申报“海上丝绸之路”世界文化遗产和丹霞地貌世界自然遗产。

丰富旅游产品。积极培育发展旅游企业，形成跨界融合的产业集团。大力发展田园风光、山水生态、美丽乡村、都市休闲、温泉养生、健康养老、文化古迹、商务会展等主题旅游产品，培育旅游消费热点。突出玉林特色，抓好旅游商品设计、生产，着力开发一批具有艺术性、实用性的旅游商品。举办旅游文化节，开展一系列旅游促销活动。深化区域旅游合作，大力拓展旅游市场。加大旅游推介力度，提高玉林旅游知名度。

优化旅游发展环境。加快景区道路、乡村旅游公路建设，促进景区道路与交通干线衔接，构建快速联动的区域旅游交通网络。加快完善旅游交通引导标识、停车场、旅游汽车营地、住宿、餐饮等旅游配套设施，创建四星级酒店3家，新建旅游集散中心3个。积极发展智慧旅游，建设旅游大数据库和旅游信息平台。加强旅游市场执法，规范旅游市场秩序，营造文明旅游大环境。

（四）积极扩大有效投资，实现重大项目建设新突破。

大力推进交通网络建设。实现玉林民用机场全面开工建设。启动建设梧州至玉林至北海城际铁路。力争上半年实现陆川通动车、年内实现玉林往广州方向开行动车目标。加快铁山港东岸码头建设，继续做好绣江复航工程前期工作。加快玉林至湛江、玉林至荔浦、松旺至铁山港东岸高速公路建设，开工浦北至北流清湾高速公路。推进普通干线公路、县乡公路、村级联网道路建设。

加快重大项目建设。贯彻落实国家、自治区系列促投资政策，采取有力措施促进投资稳定增长，年内完成固定资产投资1650亿元以上。其中，公路、铁路、港口、水利、能源等基础设施投资108亿元，教育、医疗、卫生、文化等公共服务投资50亿元，农业投资44亿元，工业投资876亿元，服务业投资80亿元。推进重大项目727个，年内完成投资466亿元，重点推进中国南药园二期、银亿新能源电池原材料生产基地、玉柴柴油机关键零部件制造与开发、兴业中广核风电场、陆川光伏发电等100个重点示范项目。

着力谋划一批项目。围绕构建完善的基础设施体系、现代产业体系、公共服务体系、生态支撑体系，深入研究和谋划一批打基础、利长远、增后劲的重大项目、重大工程，力争列入国家、自治区规划建设。多措并举破解耕地占补平衡瓶颈，确保重大项目用地。放宽民间资本准入条件，引导民间资本参与投资基础设施、产业、生态环保等领域，力争完成民间投资1400亿元。

（五）加快新型城镇化步伐，构建城市建设新面貌。

提升城市功能品质。大力推进宜居城市建设。推进城市总体规划修编，统筹城市发展空间，完善城市功能布局，提升城市品位。推进玉林城区与北流市区基础设施、路网、生态、产业等对接，加快玉北同城化发展步伐。加快推进火车站站前广场、城北汽车站、城南客运站、城东汽车站等城市交通设施，以及二环西路、三环东路等30条城市道路建设，完善城市交通网络。深入推进园博园、玉东湖二期和城区九大公园建设提升，推进海绵型道路、广场建设，提高城市排水、防涝、防洪和防灾减灾能力，加快创建海绵城市。推进智慧城市19个重点项目建设，建成智慧城市展示厅。深入开展城市交通秩序、市容环境、市场秩序专项整治，广泛开展群众性精神文明创建活动，力争成功创建自治区文明城市。

加强城市管理。深入实施《玉林市城市市容和环境卫生管理条例》，加大宣传，提高群众知晓率和参与度，落实监管责任，创新城市管理体制机制，创造和维护整洁、优美、文明的城市环境。建立完善市、县、镇三级数字化城市管理平台，推进网格化管理，实现城市管理全方位覆盖。加强社区规范化建设，提升社区服务功能，引导和发动群众积极参与城市管理。大力发展城市公共交通，鼓励大型交通企业以合资合作方式参与城市公共交通事业，增加公交线网密度，提高公交通达率。继续深化“五乱”和“四车”治理。坚决遏制违规违法建设。深化城市管理执法体制改革，整合城市管理执法职权，推动城市管理执法工作向基层延伸。

促进城镇协调发展。实施大县城战略，把基础条件好、发展潜力大的县城发展成为中小城市。贯彻落实自治区“百镇扶持壮大工程”，每个县打造1－3个中心镇。统筹推进县改市（区）、镇改办、村改居工作。深化户籍制度改革，落实居住证制度，有序推进农民工市民化，促进城镇基本公共服务常住人口全覆盖。推动城镇基础设施、产业发展、公共服务向农村延伸，社会保障向农村覆盖，促进城乡一体化发展。

（六）深化重点领域改革，激发体制机制新活力。

推进供给侧结构性改革。坚持供给和需求两侧协同发力，推动去产能、去库存、去杠杆、降成本、补短板，提高供给体系质量和效率。加快建立“僵尸企业”数据库，积极稳妥推进处置工作；继续落实房地产调控措施，促进房地产健康稳定发展；调整完善35条降成本措施，力争为企业直接减负20亿元左右；健全完善政府性债务管理体系，积极防范金融风险；补齐基础设施、公共服务、生态、民生等短板，满足群众多样化需求。

推进投融资体制改革。积极开展股权融资、产权交易融资、债券融资等多样化融资模式。推广运用政府和社会资本合作PPP模式，重点推进城市地下综合管廊工程、路网升级改造工程。引导设立各类投资基金、产业基金和合作发展基金。继续推进农村信用社改制成农村商业银行。大力引进各类新型金融机构，支持村镇银行加快发展，丰富和完善小额贷款公司，加强融资担保体系建设，新增贷款100亿元以上。

推进重点领域改革。稳步推进农村改革试验区、北流农村集体经营性建设用地入市和土地征收制度改革试点。深化农村产权制度改革，开展农村承包地“三权分置”改革试点。完善市县镇村“四级”农村产权流转交易市场体系，推进农村产权抵押融资。做好不动产统一登记工作。完善国有企业法人治理结构，健全派驻监事会管理制度，加强对国有企业的监管，加快国有企业资源整合，提高国有资本配置效率，实现国有资本保值增值，做大做强做优国有企业。积极稳妥发展混合所有制经济。全面推进国有林场、供销合作社综合改革和价格机制改革。

（七）提升开放合作水平，打造区域合作新高地。

深化全方位开放合作。主动融入“一带一路”战略，推进中泰（玉林）旅游文化产业园、中国-文莱（玉林）健康产业园规划建设。主动融入北部湾经济区开放开发，积极推进西江经济带基础设施建设大会战，加快龙港新区龙潭产业园、中医药健康产业园等重大园区建设。深化与长三角、珠三角、港澳台、京津冀等地区的合作，加快海峡两岸农业合作试验区、九洲江粤桂合作示范区建设，积极对接先进生产力。创新展会常办常新机制，进一步提升开放合作平台品牌。

加大精准招商力度。围绕新兴产业、先进制造、现代服务业、现代农业和基础设施等重点领域，实施“精准招商三年行动计划”，把长三角、珠三角、港澳台、京津冀作为主战场，把国家科研院所和高等院校技术转化、技术孵化、技术产业化以及人才交流作为主渠道，把推动企业上市，推动本土企业与世界500强、中国500强进行战略合作，培育总部经济作为主抓手，把药博会、中国-东盟小商品交易中心、中国-东盟康美（玉林）中药材交易中心等作为主平台，全方位、多视角、多角度扩大开放，努力引进一批大企业大集团。大力推进签约项目落地建设，使招商项目尽快转化为现实生产力。

发展更高层次的开放型经济。实施“引资强贸”工程，提升利用外资规模和水平。实施第二轮“加工贸易倍增计划”，推动加工贸易产业集群发展。积极参与国际产能合作，推动机械、陶瓷、现代特色农业等优势产业走出去。加快建设八角、竹木草制品、沙田柚国家级出口农产品（食品）质量安全示范区。推进广西CEPA先行先试工作。加快玉林口岸申报工作，推动通关一体化和贸易便利化。

（八）持续推进绿色发展，建设生态美丽新玉林。

大力发展生态经济。加快中滔环保产业园建设，积极培育发展节能环保产业。推进传统产业生态化升级改造，大力发展循环经济，打造新型装备制造示范基地、“城市矿产”再生资源循环产业基地。改造提升传统养殖业，大力推广“高架网床+益生菌”等生态养殖模式，力争规模养殖推广率达50%。加快推进陆川、容县国家畜牧业绿色发展示范县建设。深入推进“五彩田园”建设，大力挖掘和发展田园文化，构建生态田园体系。推进玉林－北流－容县生态景观带建设，加快形成一批生态旅游基地。

推进宜居乡村建设。在继续推进“清洁乡村”“生态乡村”活动的基础上，启动“宜居乡村”建设，重点开展“产业富民”“服务惠民”“基础便民”三个专项活动。按照“一村一品”“一镇一业”的要求，倾力打造20个“产业富民”示范村屯，辐射带动乡村产业发展。积极发展农村文化、教育、医疗等社会事业，打造30个“服务惠民”市级示范项目，提高农村居民生活保障水平。大力完善乡村基础设施，开展基础薄弱村级组织阵地攻坚建设，推进农村基层组织服务场所进步过硬，继续抓好“六个一”工程，建设40个“基础便民”村屯，加快完善农村交通、水利、环卫、信息等基础设施，加强农村污染整治和生态建设，开展生态文明示范村镇创建活动，打造一批生态文明示范村镇，实现基础设施配套完善，村容村貌整洁优美，农村人居环境质量大幅提升。

加强生态环境保护治理。深入实施“水十条、气十条、土十条”，加强水体、大气、土壤污染防治，全面提高城乡环境质量。严格耕地保护制度，划定永久基本农田并加以保护。依法打击非法采砂、采石、采矿行为，规范矿产资源开发秩序。全面落实“河长制”，加强九洲江、南流江、北流河等重点流域环境污染综合整治和生态修复建设，确保河流水质达到地表水Ⅲ类标准。开展水泥、制革、服装水洗、陶瓷、制药、造纸以及涉危涉重等行业专项清理整顿，确保企业达标排放。抓好中央环境保护督察反馈意见突出问题整改，确保按期完成整改验收。加强环境风险隐患排查整治，做好环保信访案件排查调处工作，保障全市环境安全。

（九）切实保障和改善民生，实现人民福祉新提升。

扎实推进精准脱贫攻坚。进一步完善贫困农户动态管理办法、扶贫项目库、涉农资金整合使用管理等扶贫体制机制，着力构建专项扶贫、行业扶贫、社会扶贫的大扶贫格局。推行“龙头企业+基地+贫困户”“合作社+贫困户”“电商平台+贫困户”等产业扶贫模式，形成“一村一品”“一镇一业”的产业格局。巩固脱贫成果，继续深化“六大脱贫攻坚工程”，努力实现13万贫困人口脱贫、136个贫困村出列、兴业县脱贫摘帽的目标。

努力扩大就业。实施更加积极的就业政策，健全公共就业服务体系，动态消除零就业家庭。加快创业孵化基地、农民工创业园等创业就业平台建设，推进创业担保贷款，落实就业创业扶持补贴。深入实施“精准脱贫·就业先行”就业脱贫工程，扎实开展贫困家庭“两后生”职业培训工作。做好高校毕业生、城镇就业困难人员等各类群体就业工作。

加快发展教育事业。开展学校建设提升工程，加大优质教育资源供给。加快发展学前教育，新建幼儿园10所，学前三年毛入园率达85%。加快推进玉州区望洲岭中学、东环小学太阳分校等一批中小学校建设，改善一批薄弱学校办学条件，力争陆川县、兴业县通过自治区义务教育均衡发展督导评估，玉州区、容县通过国家评估，九年义务教育巩固率达94%。开工建设福绵高中迁建工程、市一中新校区等项目，加快玉林师院附中新校区建设，高中阶段教育毛入学率达82%。推进市职业教育园区规划建设，加快广西医科大学玉林校区建设，支持玉林师范学院建设地方应用型高水平大学。推进特殊教育发展。建立健全资助体系，实现家庭经济困难学生资助全覆盖。注重学生素质教育，加强学校安全管理。加强教师队伍建设，全面提升教师队伍素质。

加快发展文化事业和文化产业。巩固国家公共文化服务体系示范区成果，完善现代公共文化服务体系。加快市图书馆新馆、档案馆新馆、科技馆、群众艺术馆新馆、非物质文化遗产展示中心、工人文化宫、王力博物馆等项目建设。全面实施文化惠民工程，延伸拓展“1小时文化圈”，打造城市“10分钟社区公共文化服务圈”。推进全民阅读，建设一批书香小镇，打造“书香玉林”。加快重要新闻媒体发展，推进传统媒体与新兴媒体融合发展。加强文化知识产权保护，保护和发展具有地方特色的优秀传统文化，打响玉林文化品牌。

推进健康玉林建设。认真贯彻“健康中国2030”规划纲要，把人民健康放在优先发展的战略地位，提升全民健康福祉。深化医药卫生管理体制改革，完善分级诊疗制度、基本药物制度和现代医院管理制度，巩固完善城市公立医院改革。推进基层医疗卫生机构能力建设行动计划，加强社区医院和村级卫生室建设，推动医疗服务重心下移和诊疗资源下沉，缓解“看病难”“看病贵”问题。完善医疗定点规划，加快建设一批医院项目。坚持计划生育基本国策。深入实施妇女儿童“两个规划”。积极应对人口老龄化，鼓励社会资本兴办健康养老服务业，推动医疗卫生和养老服务相结合。大力发展体育事业，广泛开展全民健身运动。

加强社会保障。加大社保扩面征缴工作力度。推进机关事业单位养老保险制度改革。深化基本医疗保险支付制度改革。深入推进全民参保登记计划、城镇居民大病保险、社保服务标准化建设三个国家级试点。加快推进社会保障“一卡通”工作。全面实行统一的城乡居民医保制度。加强社会救助工作，健全重特大疾病救助和疾病应急救助制度。办好社会福利事业，健全扶残助残服务体系。改进城乡低保和农村五保工作。提高住房保障水平，抓好棚户区改造、水库移民新村的规划建设。

持续推进为民办实事工程。开展社保、健康、教育、水利、安居、农补、生态、文化、扶贫、交通等十大类为民办实事工程项目，做到事事有着落，件件有回音，让广大人民群众得到更多实惠。

（十）加强社会治理能力建设，开创团结和谐新局面。

加强社会服务。加快城乡社区规范化建设，积极推进网格化管理和精细化服务，不断增强城乡社区自治和服务功能。加快培育发展行业协会、商会等各类社会组织，推动社会组织依法参与公共服务、社会管理、社区服务、公益慈善等活动。大力发展志愿服务组织。发挥工会、共青团、妇联等群团组织在社会治理中的桥梁纽带作用。探索推进政府购买服务，提高公共服务水平。

健全公共安全体系。深化平安玉林建设，完善中心城区“天网工程”和乡镇高清视频监控系统，构建立体化社会治安防控体系。加强禁毒工作，建设吸毒特殊人员监管场所。严密防范和严厉打击违法犯罪活动，强化反恐维稳工作，加大区域性火灾隐患整治，不断提升人民群众的安全感满意度。强化安全生产红线意识，完善和落实安全生产责任和管理制度，加大监管执法力度，提高安全生产监管水平。持续开展重点行业、重点领域隐患排查治理，防范重特大事故发生。完善食品药品监管体系，加强食品药品检测检验能力、基层监管能力建设，积极创建广西食品安全城市。加强防震、气象、防灾减灾救灾能力建设。完善应急管理体制，加强应急救援队伍建设，提高应急处置能力。健全社会舆情引导机制，强化互联网、无线电网络管理和安全。

加强社会治理创新。完善和落实社会稳定风险评估机制，坚决守住不发生系统性、区域性风险的底线。健全社会矛盾纠纷预防化解机制，加强信访网络信息平台建设，加大信访积案和社会矛盾化解力度。加快社会信用体系建设，构筑诚实守信的社会环境。全面实施“七五”普法规划，加强法治宣传教育，构建覆盖城乡的公共法律服务体系。积极适应军队改革发展的新形势新要求，加强国防动员和后备力量建设，深化双拥共建工作，深入推动军民融合发展。

各位代表，面对新形势、新任务，我们将以新发展理念为引领，始终牢记全心全意为人民服务的宗旨，践行忠诚、干净、担当的要求，不断提高驾驭经济、管理社会、推进改革、服务群众的能力，建设为民、务实、清廉的政府。强化担当精神，增强政治意识、大局意识、核心意识、看齐意识，加强作风和能力建设，严肃整治不作为、慢作为、乱作为等庸政懒政怠政行为，健全并严格执行工作责任制和督查问责机制，完善激励机制和容错纠错机制，激励想干事的人，保护敢干事的人，让想干事者有奔头，让敢干事者没顾虑，让干成事者得重用。强化服务意识，加快权责清单制度建设，深化“放、管、服”改革，加强事中事后监管，加快推行联合审批、网上审批，推进公共资源交易平台建设，完善服务企业机制、企业问题会商及投诉办理机制，大力开展“互联网+益民服务”行动，让信息多跑路、群众少跑腿。强化法治思维，全面落实法治政府建设实施纲要，大力推进依法治市，严格按照法定权限、法定程序行使职权，推进重大决策评估，严格执行市人大及其常委会决定，自觉接受市人大及其常委会依法监督和市政协民主监督，认真听取人大代表、政协委员和各民主党派、工商联、人民团体的意见建议，提高人大代表建议和政协委员提案办理质量。主动接受社会监督和舆论监督，让人民群众更有效地监督政府。强化纪律规矩观念，严格落实全面从严治党主体责任，全面加强和规范党内政治生活，持续提升政府系统从严管党治党水平。深化落实中央“八项规定”精神，巩固“两学一做”学习教育成果。加强重点领域审计监督，严格控制“三公”经费支出。运用监督执纪“四种形态”，严厉查处群众身边特别是扶贫领域的不正之风和腐败问题，以坚定的意志和决心反腐惩恶，以反腐惩恶的成效保障发展、取信于民，营造风清气正的政治生态。

各位代表，担当书写发展，实干成就梦想。让我们更加紧密团结在以习近平同志为核心的党中央周围，在市委的坚强领导下，解放思想，勇于担当，牢记使命，实干兴玉，全面夯实“两个建成”的坚实基础，以优异成绩迎接党的十九大胜利召开！
